# Gamma Oryzanol Alleviates High-Fat Diet-Induced Anxiety-Like Behaviors Through Downregulation of Dopamine and Inflammation in the Amygdala of Mice

**DOI:** 10.3389/fphar.2020.00330

**Published:** 2020-03-17

**Authors:** Salina Akter, Kazi Rasel Uddin, Hiroyuki Sasaki, Shigenobu Shibata

**Affiliations:** Laboratory of Physiology and Pharmacology, School of Advanced Science and Engineering, Waseda University, Tokyo, Japan

**Keywords:** high-fat diet, γ-oryzanol, anxiety, dopamine, amygdala

## Abstract

**Background:**

A high-fat diet (HFD) can induce obesity and metabolic disorders that are closely associated with cognitive impairments, and the progression of several psychiatric disorders such as anxiety. We have previously demonstrated the anxiolytic-like effect of Gamma oryzanol (GORZ) in chronic restraint stressed mice.

**Objective:**

We studied the neurochemical and molecular mechanisms that underlie the preventive effect of GORZ in HFD-induced anxiety-like behaviors, monoaminergic dysfunction, and inflammation.

**Methods:**

Eight-week-old Institute of Cancer (ICR) male mice weighing 33–34 g were divided into the following groups and free-fed for 8 weeks: control (14% casein, AIN 93M); HFD; HFD + GORZ (0.5% GORZ). Body weight gain was checked weekly. The anxiolytic-like effects of GORZ were examined *via* open-field test (OFT) and elevated plus maze (EPM) test. Brain levels of monoamines [5-hydroxy tryptamine (5-HT), dopamine (DA), and norepinephrine (NE)] and their metabolites [5-hydroxyindole acetic acid (5-HIAA), homovanillic acid (HVA), and 3-methoxy-4-hydroxyphenylglycol (MHPG)], proinflammatory cytokines such as tumor necrosis factor-*α*α (*Tnf-α*) mRNA levels, and interleukin 1-β (*Il-1β*) mRNA levels in the cerebral cortex and amygdala were examined using high-performance liquid chromatography-electrochemical detection (HPLC-ECD), and real-time reverse transcription-polymerase chain reaction (RT-PCR), respectively.

**Results:**

Mice fed a HFD for eight weeks showed anxiety-like behaviors in association with HFD-induced body weight gain. GORZ potentially blocked HFD-induced anxiety-like behaviors *via* significant improvement of the primary behavioral parameters in behavioral tests, with a minor reduction in HFD-induced body weight gain. Furthermore, GORZ treatment significantly downregulated HFD-induced upregulation of dopamine levels in the brain's amygdala. Significant reduction of the relative mRNA expression of *Tnf-α* and *Il-1 β* was also observed in the amygdala of HFD + GORZ mice, compared to HFD mice.

**Conclusions:**

Our findings strongly suggest that 0.5% GORZ exerts anxiolytic-like effects, possibly through downregulation of dopamine, and *via* expression of proinflammatory cytokines *Tnf-α* and *Il-1 β* in the case of chronic HFD exposure.

## Introduction

Long-term consumption of a high-fat diet (HFD) affects whole-body homeostasis and contributes to the development of weight gain/obesity and associated comorbidities, including depression and anxiety (Anderson et al., 2001; Petry et al., 2008; Gadalla, 2009; Mizunoya et al., 2013; Jacka et al., 2015). Several reports suggest that chronic HFD exposure also leads to the progression of successive levels of inflammation (De Souza et al., 2005; Stienstra et al., 2011). In fact, inflammation partially arises due to an influx of macrophages that secrete proinflammatory cytokines such as tumor necrosis factor-alpha (TNF- **α) and interleukin-1 beta (IL-1β) (Hotamisligil et al., 1993; Kanda et al., 2006; Todoric et al., 2006). Dutheil et al. showed that a 4-month HFD caused anxiety and anhedonic behaviors in male Sprague-Dawley rat models, associated with increased expression of inflammatory cytokines (Dutheil et al., 2016).

Altered central monoaminergic functions have been implicated in the pathophysiology of anxiety. It is well-documented that HFD-induced behavioral impairments are closely associated with alteration of brain neurochemistry in a region-specific manner (Molteni et al., 2004; Sharma and Fulton, 2013), partially caused by a diet high in fat. For example, a chronic (3 months) HFD altered striatal and mesolimbic dopamine (DA) signaling in rodents (Davis et al., 2008; Sharma and Fulton, 2013). Additionally, another animal study reported that chronic HFD consumption for 10 weeks targeted the cortex of mice, and caused emotional disturbances *via* DA dysfunction, characterized by increased DA turnover (Wakabayashi et al., 2015).

Gamma oryzanol (GORZ), preliminarily extracted from rice bran oil, is an important bioactive component comprising a blend of trans-ferulic acid esters (trans-hydroxycinnamic acid) and phytosterols (sterols and triterpenic alcohols), such as cycloartenol, β-sitosterol, 24-methylenecycloartenol, and campesterol (Xu and Godber, 1999; Lerma-García et al., 2009). It can also be found in lesser amounts in barley, maize, wheat, oats, asparagus, tomatoes, peas, berries, olives, vegetables, fruits (particularly citrus fruits), and several other foods. It has been extensively used as an antioxidant, antiulcerogenic, antineoplastic, antidiabetic, and antiallergic drug for many years (Islam et al., 2011; Kim et al., 2012; Kozuka et al., 2013; Goufo and Trindade, 2014; Kozuka et al., 2015). Therapeutic use of GORZ has also been approved for inflammation and nervousness (Chotimarkorn and Ushio, 2008; Araujo et al., 2015). In addition, GORZ can decrease the risk of HFD-induced obesity by inhibiting HFD-induced hyperlipidemia and oxidative stress in mice (Jin Son et al., 2010), although very little is known about the molecular mechanisms involved in this process.

Recent data from our existing research suggest the anxiolytic mechanism of GORZ as being related to brain levels of monoamines in the amygdala of the mouse brain, as observed in chronic restraint stressed mice (Akter et al., 2019). However, the antiinflammatory effect of GORZ, alongside its anxiolytic role, have to date not been analyzed. Thus, we established an HFD-induced anxious mice model in the present study, with the aim of investigating the neurochemical and molecular mechanisms underlying the preventive effect of GORZ on HFD-induced anxiety-like behaviors, monoaminergic dysfunction, and inflammation.

## Methods and Materials

### Animals

**Figure 1:**
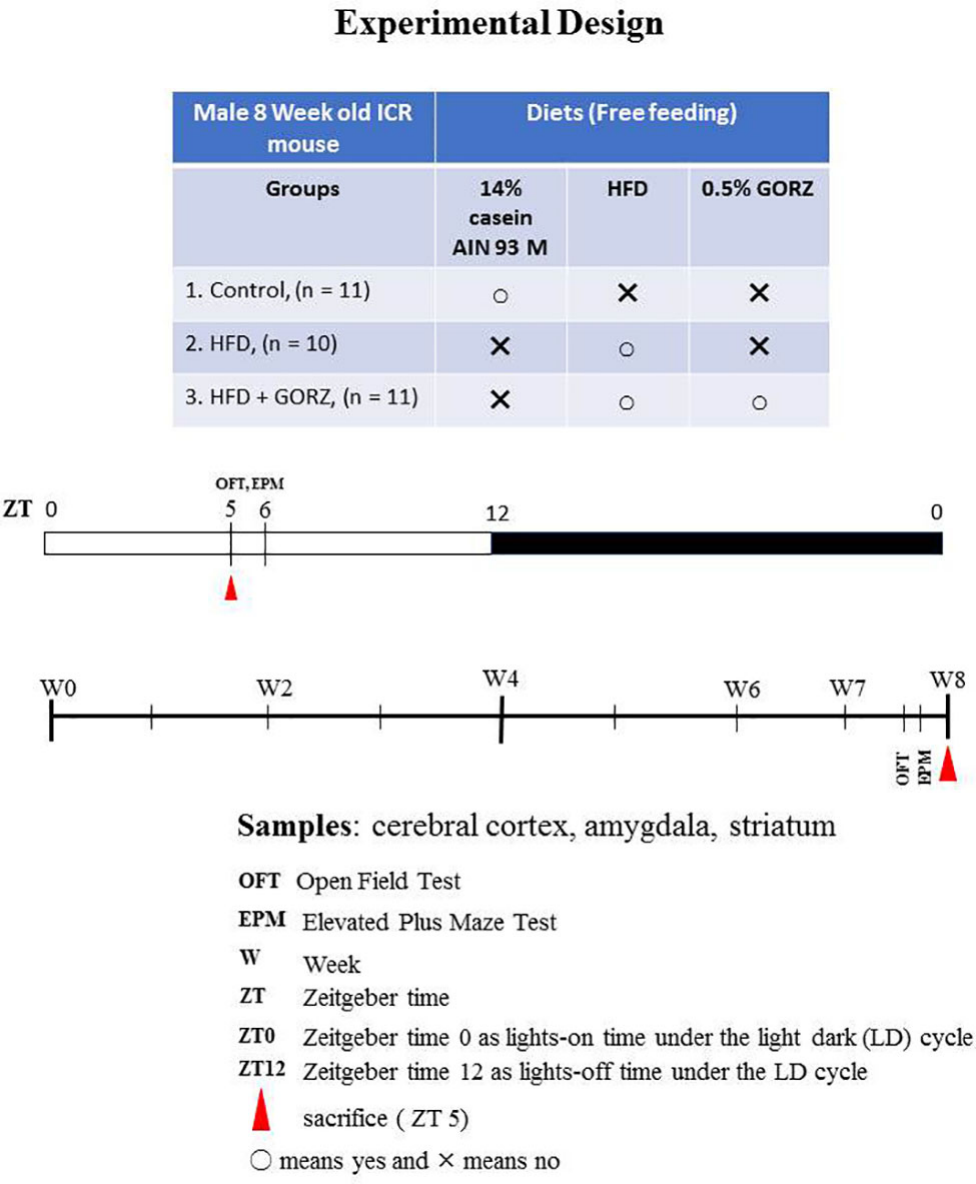
Schematic representation of the experimental design and protocol.

Eight-week-old Institute of Cancer Research (ICR) male mice were purchased from Tokyo Laboratory Animals Science Co., Ltd. (Tokyo, Japan). **Figure 1** illustrates the experimental design and protocol. Mice were weight-matched and randomly divided into three groups: a control, HFD, and HFD + GORZ group. Animal experiments were performed under standard conditions, during which a 12:12 light/dark cycle (with lights on at 8:00) was observed, accompanied by a constant temperature and humidity environment (22°C ± 2°C, 60% ± 5%). Zeitgeber time (ZT) 0 and 12 represented times when lights were on and off, respectively. Food and tap water were available *ad libitum*. All animal care procedures conformed to the “Fundamental Guidelines for Proper Conduct of Animal Experiment and Related Activities in Academic Research Institutions” (published by the Ministry of Education, Culture, Sports, Science and Technology, Japan, June 1st, 2006), and were approved by the Committee for Animal Experiments at Waseda University, Japan (permission #2017-A074).

### Treatments

The composition of different diets fed to mice are listed in **Table 1**. The control diet and HFD were obtained from Oriental Yeast Co. Ltd. (Tokyo, Japan). For preparation of the HFD + GORZ food, GORZ was obtained from Sigma-Aldrich (St. Louis, Missouri, USA). A GORZ ratio of 0.5% was selected based on our existing study (Akter et al., 2019), fixed with the weight of HFD, and administered to mice. Mice were allowed free access to food and tap water during the experimental period, and exposure time was the same for all groups of mice. Food and tap water were changed once a week; mice were transferred to new cages with fresh wood shavings weekly. Following the suggestion of the animal etiological committee affiliated by Waseda University, 10 to 11 mice were group housed in each cage as one treatment group, each representing an experimental unit. Mice were thus observed in groups in terms of food consumption and as such, individual mouse data related to food consumption was not recorded. Body weight gain was monitored weekly. The general health status of each mouse was checked and maintained at a satisfactory level. Additionally, a HFD was administered chronically and all behavioral tests were conducted while animals were maintained on a HFD, and not in a state of withdrawal.

**Table 1:** The compositions of different diets fed to mice.

Nutrition	Diet			
	Control (14% casein AIN-93 M)		HFD	
	gm%	Kcal%	gm%	Kcal%
Protein	14	15	24	20
Carbohydrate	73	76	41	35
Fat	4	9	24	45
Total		100.0		100.0
Kcal/gm	3.8		4.73	
**Ingredient**	gm	Kcal	gm	Kcal
Casein	140	560		
Casein, 30 Mesh			200	800
L-Cysteine	1.8	7	3	12
Corn starch	495.692	1,983	72.8	291
Maltodextrin	125	500	100	400
Sucrose	100	400	172.8	691
Cellulose, BW200	50	0	50	0
Soybean oil	40	360	25	225
t-butylhydroquinone	0.008	0	0	0
Lard	0	0	177.5	1,598
Mineral Mix S10022M	35	0	0	0
Mineral Mix S10026	0	0	10	0
Dicalcium Phosphate	0	0	13	0
Calcium Carbonate	0	0	5.5	0
Potassium Citrate, 1 H_2_O	0	0	16.5	0
Vitamin Mix V10001	0	0	10	40
Vitamin Mix V10037	10	40	0	0
Choline Bitrate	2.5	0	2	0
FD & C Red dye # 40			0.05	0
**Total**	**1,000**	**3,850**	**858.15**	**4,057**

### Behavioral Tests

To examine the anxiolytic-like effects of 0.5% GORZ, two behavioral tests were performed between ZT 5 and 6 in the following order: the open-field test (OFT) and an elevated plus maze (EPM) test using light intensity of 100–150 lux (**Figure 1**). The interval between the two behavioral tests was one day (24 h), in order to prevent any possible interference.

#### Open-Field Test

An OFT is one of the most commonly used platforms for assessing anxiety-like behaviors among mice (Lau et al., 2008). On week 7 and day 4, mice were subjected to the OFT at ZT 5 to 6. For OFT, each mouse was placed at the center (23*12 cm) of the open-field apparatus (35*24*24 cm). A charged-coupled device (CCD) camera was used to record behavior for a 5-min period. Several important parameters including time in the center zone (s), total distance traveled (m), and number of entries into the center zone were measured to assess anxiety-like behavior, and analyzed using ANY-maze software (version 4.99, Stoelting, IL, USA). The apparatus was thoroughly cleaned with 70% ethanol after each OFT trial.

#### EPM Test

Following the one day interval period after the OFT, anxiety-related behavior of mice was assessed by another frequently used behavior assay, the EPM test, on week 7 and day 5, at ZT 5 to 6. In EPM, a square center zone (7 cm) was connected by two opposite open arms (29 × 7 cm) and two opposite closed arms (29*19*7 cm) which was elevated approximately 41 cm above the floor. An appropriate observation room was also fixed to locate the EPM. The walls on the closed arms were opaque. Each mouse was placed at the center zone facing the open arm and was allowed to move freely for a 5-min period. A CCD camera was used to record the 5-min EPM test. Important parameters such as the time (s), distance traveled (m), and entry number for both open and closed arms were calculated and analyzed, using ANY-maze software (version 4.99,　Stoelting, IL, USA). ANY-maze detected arm entries using the mouse body center as reference for the location of the mouse. The apparatuses were cleaned with 70% ethanol after each trial in the EPM test.

### Animal Sacrifice and Sample Collection

Following the 2-day interval period after the EPM test, mice were sacrificed at ZT 5 to 6 at the end of 8 weeks' chronic dietary treatment (**Figure 1**). Midazolam (4 mg/Kg, i.p.)/xylazine (10 mg/Kg, i.p.) was used to anesthetize mice and trunk blood samples were collected following decapitation. Serum was obtained by keeping the samples at room temperature for 1 h, centrifuged at 3,000 rpm for 15 min, and stored at –80°C until further analysis. Whole brains were also rapidly removed. Frontal brain slices (2 mm thick) including the cerebral cortex and amygdala were prepared using a brain matrix (# 0530; Bioresearch Center Co., Nagoya, Japan). The amygdalae (mean wet weight, 11.49 mg) were dissected free-hand. The cerebral cortices (mean wet weight, 14.55 mg) were dissected free-hand from the upper regions of the dorsal hippocampus. Detailed protocol for slicing and dissecting brain areas of interest according to the brain atlas (from bregma –0.5 to bregma –2.5) is described in our previous paper (Akter et al., 2019). Brain tissue was moved to a –80°C freezer and stored at –80°C until further analysis.

### High-Performance Liquid Chromatography-Electrochemical Detection

To explore the HFD stress-associated neurochemical mechanisms involved in the anxiolytic-like effects of 0.5% GORZ, 5-hydroxytryptamine (5-HT), DA, and norepinephrine (NE), alongside their metabolites, 5-hydroxyindole acetic acid (5-HIAA), homovanillic acid (HVA), and 3-methoxy-4-hydroxyphenylglycol (MHPG) were detected in the cerebral cortex and amygdala, using high-performance liquid chromatography-electrochemical detection (HPLC-ECD) (HTEC 500; Eicom, Kyoto, Japan), following behavioral assessments. Samples for 5-HT, DA, and NE, alongside measurement of their metabolites, received 0.2 M perchloric acid (including 100 μM EDTA·2Na), containing 20 ng of isoproterenol (internal standard). Samples were homogenized using an ultrasonic-homogenizer prior to being centrifuged at 15,000 rpm at 4°C for 15 min. The supernatant for each sample was collected and filtered using a 0.45-μm filter. HPLC-ECD was used to measure and quantify monoamine in each 20 μl sample. During HPLC-ECD, the following conditions were maintained: 85% of the transfer phase was composed of a 0.1 M acetate citric acid buffer (pH 3.5), including 5 mg/L EDTA·2 Na, 190 mg/L 1-octanesulfonic acid sodium salt, and 15% methanol. The velocity of flow was 500 μl/min. The applied column temperature and voltage were 25°C and + 750 mV vs. Ag/AgCl, respectively. EPC-300 software (version 2.5.10, Eicom) was used to evaluate the data. The applied protocol is published in detail in our existing paper (Akter et al., 2019).

### Real-Time Reverse Transcription-Polymerase Chain Reaction

Relative mRNA levels of neuroinflammatory genes *Tnf-α* and *Il-1β* were measured in the cerebral cortex and amygdala by quantitative real-time reverse transcription-polymerase chain reaction (RT-PCR), using the One-Step SYBR RT-PCR Kit (Takara Bio Inc., Shiga, Japan) with specific primer pairs (see **Supplementary Table 1**, online) and a Piko Real PCR system (Thermo Fisher Scientific, Waltham, MA, USA). Total RNA was extracted using the TRIzol Reagent (Ambion, Oakland, USA) as per the manufacturer's instructions. Total RNA yield was evaluated by NanoVue Plus (GE Healthcare Life Sciences, Piscataway, NJ, USA). The primers were designed using Primer 3 software version 4.1.0 (Koressaar and Remm, 2007; Untergasser et al., 2012). Real-time reverse transcription-polymerase chain reaction was carried out under the following conditions: cDNA synthesis at 42°C for 15 min, followed by 95°C for 2 min, PCR amplification for 40 cycles with denaturation at 95°C for 5 s, and annealing and extension at 60°C for 20 s. The relative expression levels of the target genes were normalized to that of *18srRNA*. Data were analyzed using the ΔΔCt method. A melt curve analysis of each primer was performed to identify nonspecific products. The detailed protocol for this process is provided in our existing paper (Tahara et al., 2015).

### Statistical Analysis

**Figure 2:**
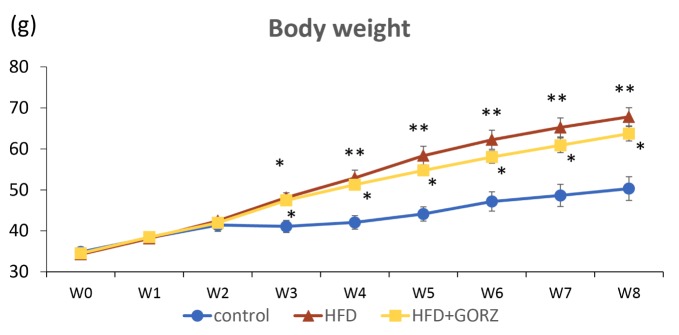
The effect of 0.5% GORZ treatment on HFD-induced body weight gain (g). Values are presented as mean ± standard error (SE); n = 10–11 mice per group. Statistical differences were evaluated by Kruskal-Wallis test with a Dunn *post hoc* analysis, and a two-stage linear step-up procedure of the Benjamini, Krieger, and Yekutieli test for multiple comparisons; *P < 0.05, **P < 0.01 vs. control.

Statistical analyses were performed using GraphPad Prism (version 6.03, GraphPad Software, San Diego, CA, USA), and data were expressed as mean ± standard error of the mean. We determined whether the data showed a normal or nonnormal distribution, and equal or biased variation using the D'Agostino-Pearson test and Bartlett's test, respectively. If the data showed normal distribution and equal variation, parametric analyses were conducted using a one-way or two-way analysis of variance (ANOVA), followed by Tukey *post hoc* analysis. If the data showed a nonnormal distribution or biased variation, nonparametric analyses were performed using a Kruskal-Wallis test, followed by the Dunn's *post hoc* test. **Tables 2** and **3** summarize the use of parametric and nonparametric analyses. The body weight data in **Figure 2** include two factors, i.e., week and group; however, the data showed nonnormal distribution or biased variation. Accordingly, we conducted nonparametric analysis using a Kruskal-Wallis test, with a Dunn's *post hoc* test and a two-stage linear step-up procedure of the Benjamini, Krieger, and Yekutieli test, in order to affect multiple comparisons. The significant mark in **Figure 2** indicates only comparisons of food within a period of one week, as we focused on assessment of food, rather than of time. Statistical differences were considered significant when P value was less than 0.05.

**Table 2:** Results of one-way ANOVA and Kruskal-Wallis behavioral tests (OFT and EPM tests).

Detail	Statistic	F	P-value
Time in the center zone	Kruskal-Wallis	–	0.4909
Total distance travelled	Kruskal-Wallis	–	0.0032
No. of entries to the center zone	Kruskal-Wallis	–	0.0114
Time in the open arm	One-way ANOVA	F (2, 29) =3.822	0.0336
Distance travelled in the open arm	One-way ANOVA	F (2, 29) =3.270	0.0424
No. of entries to the open arm	Kruskal-Wallis	–	0.2656
Time in the closed arm	One-way ANOVA	F (2, 29) =10.46	0.0004
Distance travelled in the closed arm	One-way ANOVA	F (2, 29) =1.362	0.2721
No. of entries to the closed arm	One-way ANOVA	F (2, 29) =1.659	0.2080

**Table 3:** Results of one-way ANOVA and Kruskal-Wallis test for central monoamines with their metabolites and inflammatory genes in the cerebral cortex and amygdala.

Detail		Statistic	F	P value
Cortex	5-HT	One-way ANOVA	F (2, 24) =2.840	0.0781
Cortex	5-HIAA	Kruskal-Wallis	–	0.2910
Cortex	DA	Kruskal-Wallis	–	0.9044
Cortex	HVA	Kruskal-Wallis	–	0.4570
Cortex	NE	One-way ANOVA	F (2, 24) =1.263	0.3008
Cortex	MHPG	One-way ANOVA	F (2, 24) = 0.2943	0.7477
Amygdala	5-HT	Kruskal-Wallis	–	0.001
Amygdala	5-HIAA	One-way ANOVA	F (2, 24) = 3.023	0.0675
Amygdala	DA	One-way ANOVA	F (2, 24) = 9.157	0.0011
Amygdala	HVA	Kruskal-Wallis	–	0.8251
Amygdala	NE	One-way ANOVA	F (2, 24) = 4.830	0.0173
Amygdala	MHPG	Kruskal-Wallis	–	0.1977
Cortex	*Tnf-α*	One-way ANOVA	F (2, 22) = 0.6618	0.5264
Cortex	*Il-1β*	One-way ANOVA	F (2, 22) = 0.3407	0.7151
Amygdala	*Tnf-α*	One-way ANOVA	F (2, 22) = 14.23	0.0001
Amygdala	*Il-1β*	Kruskal-Wallis	–	0.0138

## Results

Initially, a preliminary behavioral study including the EPM test was conducted using 5-week-old ICR male mice. The mice were randomly divided into two groups, a control (14% casein diet, n = 11) and a 0.5% GORZ group (14% casein diet + 0.5% GORZ, n = 12), and housed for 4 weeks with *ad libitum* food and tap water, under a 12-h light/dark cycle. For the EPM test, in the open arm, important behavioral parameters such as time spent (s), distance traveled (m), and the number of entries in the control and 0.5% GORZ mice groups were 58.79 ± 7.65, 0.79 ± 0.12, and 15.45 ± 1.71, respectively, and 48.65 ± 5.77, 0.74 ± 0.13, and 15.58 ± 1.44, respectively. Concurrently, in the closed arm of the EPM test, time spent (s), distance traveled (m), and the number of entries in the control and 0.5% GORZ mice groups were 174.38 ± 12.03, 4.53 ± 0.36, and 22.81 ± 2.2, respectively, and 177.68 ± 9.27, 4.50 ± 0.31, and 23.58 ± 1.02, respectively. A lack of significant changes in terms of important behavioral parameters recorded by the EPM test in this preliminary experiment inspired us to formulate the present study design with three groups: control, HFD, and HFD + GORZ.

### Effects of GORZ on Body Weight Gain

Initially, body weight did not vary significantly among the three groups of mice up to a period of 2 weeks. Body weight gains significantly increased at each time point from 3 weeks onwards up to 8 weeks, and was higher in both HFD and HFD + GORZ groups, compared to the control group (**Figure 2**). Body weight gain in the HFD + GORZ group remained lower than in the HFD group from week 3 until the end of the 8-week experiment, but the difference was not significant (**Figure 2**).

### Effects of GORZ on Chronic HFD-Induced Anxiety-Like Behaviors in OFT

**Figure 3:**
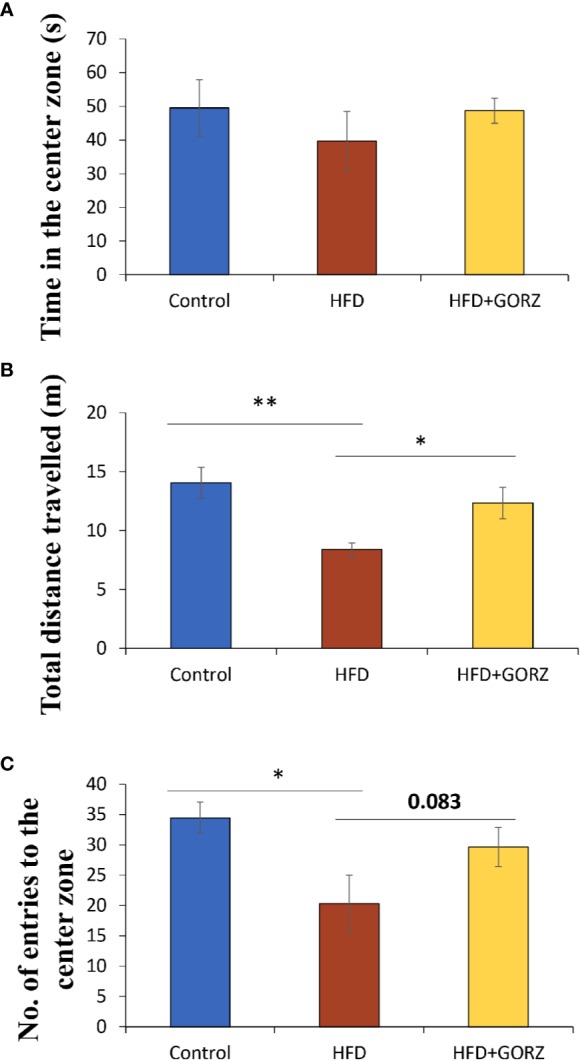
The effect of 0.5% GORZ treatment on HFD-induced anxiety in the open-field test (OFT): **(A)** time in the center zone (s); **(B)** total distance traveled (m); **(C)** the number of entries into the center zone. Values are presented as mean ± standard error (SE); n = 10–11 mice per group. Statistical differences were evaluated by Dunn's test; * P< 0.05, **P < 0.01.

Anxiety-related behavior in the OFT is illustrated in **Figure 3**. In the OFT, time in the center zone (s), total distance traveled (m), and number of entries into the center zone were impacted in the HFD group. The total distance traveled (m) was significantly lower among HFD mice than in control mice, thereby promoting chronic HFD-induced anxiety-like behavior (**Figure 3B**). In addition, mice treated with HFD + 0.5% GORZ displayed a significant increase in total distance traveled (m), compared to HFD-treated mice (**Figure 3B**). Furthermore, HFD-induced anxiety-like behavior was also confirmed, as the number of entries into the center zone was significantly decreased in HFD mice, compared to control mice (**Figure 3C**). Moreover, compared to the HFD group, mice treated with 0.5% GORZ showed an increased trend in the number of entries into the center zone (P = 0.083), due to the anxiolytic-like effects of GORZ (**Figure 3A**). Although significant differences were not observed among the mice groups, 0.5% GORZ treatment marginally reversed the HFD-induced decrease in time in the center zone (s) (**Figure 3**). The results of one-way ANOVA and Kruskal-Wallis test for each evaluation are shown in **Table 2**.

### Effects of GORZ on Chronic High-Fat Diet-Induced Anxiety-Like Behaviors in the EPM Test

**Figure 4:**
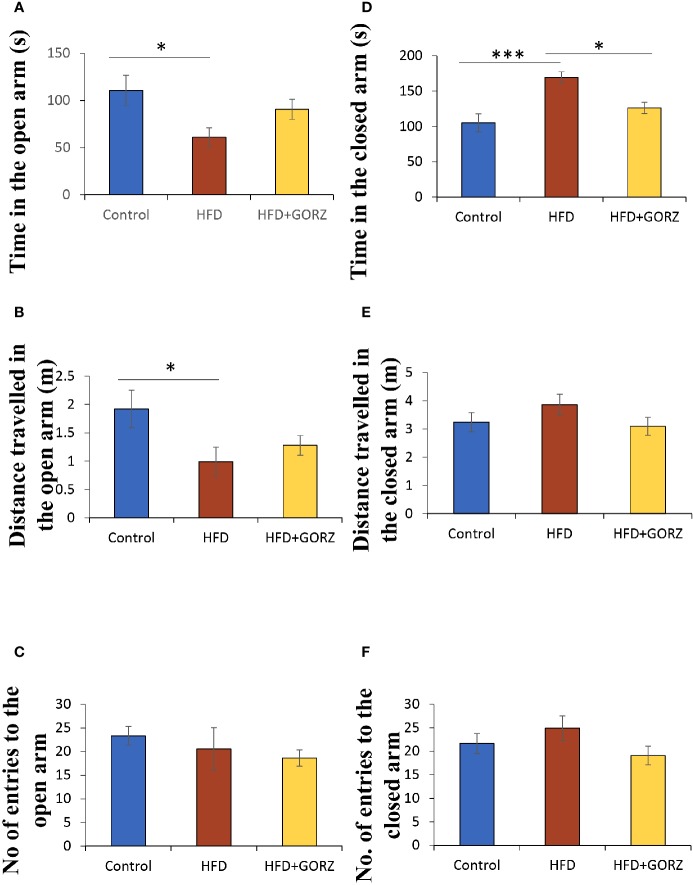
The effect of 0.5% GORZ treatment on HFD-induced anxiety in the elevated plus maze (EPM) test: **(A)** time in the open arm (s); **(B)** distance traveled in the open arm (m); **(C**) number of entries into the open arm; **(D)** time in the closed arm (s); **(E)** distance traveled in the closed arm (m); **(F)** number of entries into the closed arm. Values are presented as mean ± standard error (SE); n = 10–11 mice per group. Statistical differences were evaluated by Tukey's test; *P < 0.05, ***P < 0.001.

Decreased time spent (s) and distance traveled (m) into the open arm, and increased time spent (s) in the closed arm among HFD-treated mice, compared to control mice, showed that HFD increased behaviors indicative of anxiety (**Figures 4A, B, D**). Remarkably, however, a 0.5% GORZ treatment was able to attenuate the HFD-induced anxiety-like symptoms, as a slight increase in time spent (s) and distance traveled (m) into the open arm, and a significant decrease in time spent in the closed arm (s) were observed in this group (**Figures 4A, B, D**). There were no significant changes in the number of entries into the open and closed arm, or distance traveled in closed arms (m) among the experimental groups in the EPM test (**Figures 4C, E, F**). The results of one-way ANOVA and Kruskal-Wallis test for each evaluation are shown in **Table 2**.

### The Effects of GORZ on the Central Monoaminergic Pathway in the Cerebral Cortex and Amygdala

**Figure 5:**
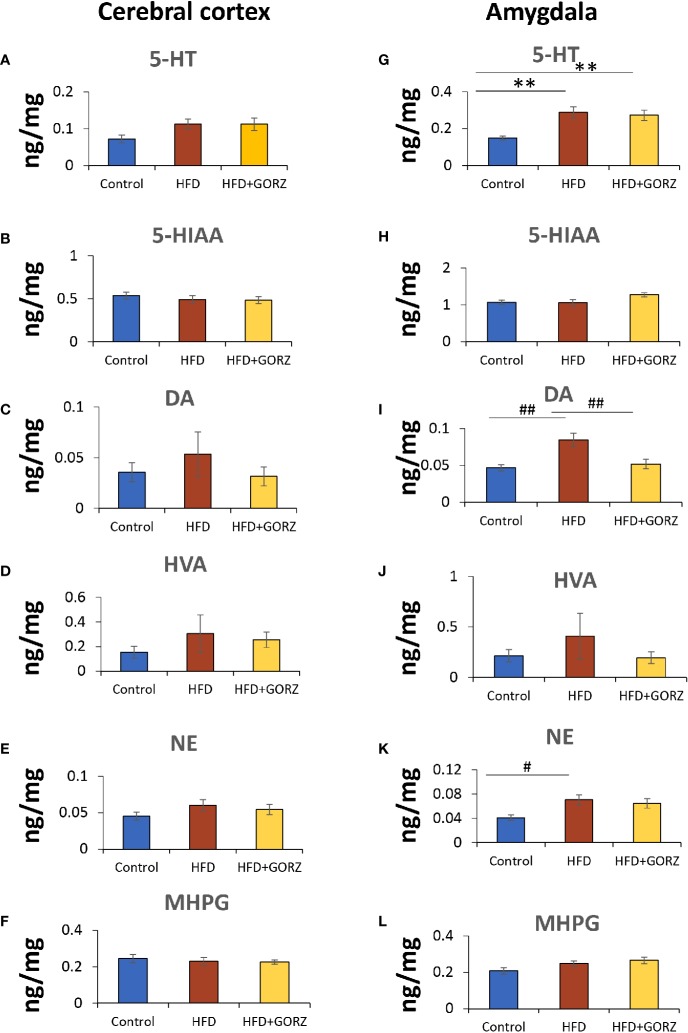
The effect of 0.5% GORZ treatment on levels (ng/mg) of central monoamines, with their metabolites in the cerebral cortex and amygdala. **(A–F)** respectively: 5-HT, DA, and NE, and their metabolites, 5-HIAA, HVA, and MHPG, respectively, in the cerebral cortex; **(G–L)** respectively: 5-HT, DA, and NE, and their metabolites, 5-HIAA, HVA, and MHPG, respectively, in the amygdala. Values are presented as mean ± standard error (SE); n = 9 mice per group. Statistical differences were evaluated by Tukey's test; ^#^P < 0.05, ^##^P < 0.01 and Dunn's test; **P < 0.01.

In this regard, 5-HT showed a significant increase both in the amygdala in HFD-fed mice compared to control mice (**Figures 5A, G**). Additionally, HFD mice also showed significantly higher DA and NE values in the amygdala but not in the cerebral cortex (**Figures 5C, E, I, K**). The most important effect of 0.5% GORZ dietary treatment was detected in the amygdala, where GORZ significantly prevented the DA upregulation observed in HFD-fed mice (**Figure 5I**). Both HVA and NE were decreased in the cerebral cortex and amygdala in the HFD + GORZ group, compared to the HFD group, although the differences were not statistically significant (**Figures 5D, E, J, K**). The metabolites such as 5-HTIAA and MHPG did not show any changes among 28 groups (**Figures 5B, F, H, L**). The results of one-way ANOVA and Kruskal-Wallis test for each evaluation are shown in **Table 3**.

### Effects of GORZ on the Expression of Inflammatory Genes *Tnf-α* and *Il-1* in the Cerebral Cortex and Amygdala

**Figure 6:**
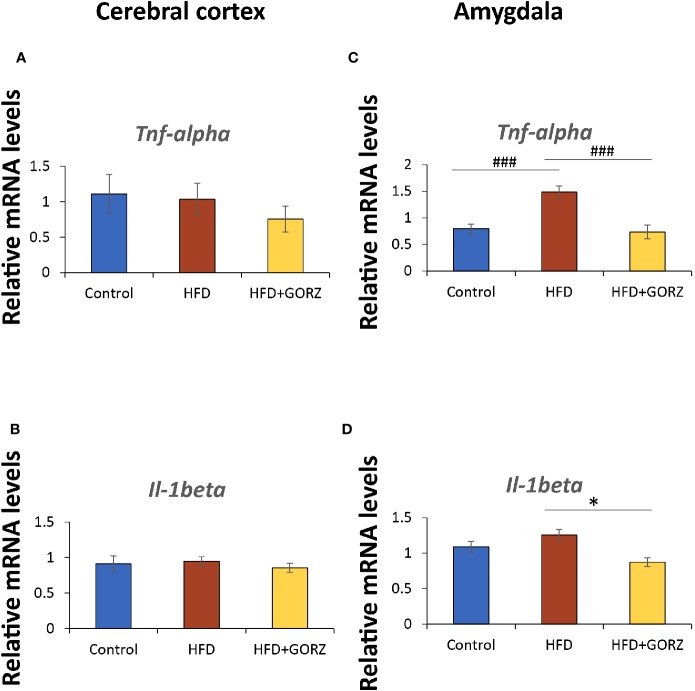
The effect of 0.5% GORZ treatment on the relative mRNA levels of proinflammatory cytokines *Tnf-α* and *Il-1β* in the cerebral cortex and amygdala. Relative mRNA levels of **(A)** *Tnf-α* and **(B)** *Il-1β* in the cerebral cortex. Relative mRNA levels of **(C)** *Tnf-α* and **(D)** *Il-1β* in the amygdala. Values are presented as mean ± standard error (SE); n = 8–9 mice per group. Statistical differences were evaluated by Tukey's test; ^###^P < 0.001 and Dunn's test; *P < 0.05.

There were no significant differences in the *Tnf-α* and *Il-1* mRNA levels among the experimental groups in the cerebral cortex (**Figures 6A, B**). However, HFD mice showed a significant increase in the amygdala's mRNA levels of *Tnf- α* compared to the control group, and this increase was significantly suppressed by a 0.5% GORZ dietary treatment

(**Figure 6C**). Furthermore, *Il-1* mRNA expression was also significantly decreased in the amygdala of HFD + GORZ mice, compared to the HFD group (**Figure 6, D**). The results of one-way ANOVA and Kruskal-Wallis test for each evaluation are shown in **Table 3**.

## Discussion

We previously demonstrated that 0.5% GORZ may act as a promising anxiolytic functional food in chronic restraint stressed mice, *via* regulation of central monoamine neurotransmitters in the amygdala (Akter et al., 2019). However, the antiinflammatory effect of GORZ, along with its anxiolytic function, also served as areas of focus in the current study. In the present study, we investigated the neurochemical and molecular mechanisms that underlie the anxiolytic-like effects of 0.5% GORZ on high-fat dietary stress.

### Chronic High-Fat Diet-Induced Anxiety-Like Behaviors Can Potentially Be Attenuated by GORZ in OFT and EPM Test

Many studies have consistently shown that a HFD increases the risk of HFD-induced stress, and can give rise to the development of neuropsychiatric disorders such as anxiety and depression (Souza et al., 2007; Sharma et al., 2012). There is substantial evidence that a HFD exacerbated depressive-like behavior in a rat model (Abildgaard et al., 2011). It has already been shown that the OFT and EPM test are widely used assays for assessing anxiety-like behaviors (Lau et al., 2008; Akter et al., 2019). In the present study, mice maintained on a HFD for 8 weeks showed strong anxiety-like behaviors, evidenced by a significant reduction in total distance traveled (m), the number of entries into the center zone in the OFT, and open arm time spent (s) and distance traveled (m) in the EPM test. A consistent study reported that HFD mice traveled less, and without differences in center preference in OFT and where increased anxiety-like behaviors were present (Gelineau et al., 2017). In our preliminary data, GORZ dissolved in AIN-93M standard food did not show any anxiety or anxiolytic-like effects in a behavioral test; this suggests GORZ is an ideal functional food, as it indicated a protective effect to HFD-induced abnormal behavior, without any effect on normal behavior.

Animals fed a HFD chronically for 8 weeks also showed significantly higher body weight compared to control mice, in conjugation with increased vulnerability to anxiety-like behaviors. Chronic 0.5% GORZ supplementation in HFD-fed mice showed a weight loss trend compared to only HFD-fed mice for 8 weeks. In summary, GORZ can potentially attenuate HFD-induced anxiety-like behaviors through significant improvement of primary behavioral parameters, as observed in the behavioral tests, with a slight reduction in HFD-induced body weight gain. Consistently, Kozuka et al. showed that brown rice-specific GORZ significantly attenuated the preference for a HFD, with an apparent reduction in HFD-induced body weight gain *via* hypothalamic regulation of endoplasmic reticulum stress in mice (Kozuka et al., 2012; Kozuka et al., 2017).

In this experimental design, a total of 10 to 11 mice were housed in each cage as a treatment group, each constituting an experimental unit. Therefore, we were not able to measure food consumption per mouse, or evaluate feeding impact in body weight change. In accordance with Animal Care Committee guidelines (March 13th, 2017), group housing was preferred to avoid stress responses and individual housing-induced anxiety-like behaviors. However, we cannot exclude the possibility that individual variations in food intake may have impacted all behavioral and biochemical analyses; an individual housing experiment may therefore be required in future. If group housing is still important, two animals could be group housed, and then this procedure would have given n=5–6.

Behavioral assessment outcomes for 0.5% GORZ-treated mice resulted in significant expansion of total distance traveled (m) in the OFT, as well as significant decreases in time (s) spent in the closed arm of the EPM test. Accordingly, the behavioral test results confirmed the hypothesis that a chronic HFD consumption for 8 weeks, as a chronic stress, caused mice to enter an anxious emotional state, and 0.5% GORZ can potentially attenuate HFD-induced anxiety-like behaviors, which supports our previous findings (Akter et al., 2019). Although we examined the GORZ-induced anxiolytic-like effect by both OFT and EPM assays to confirm reproducibility, other behavioral assay by conflict paradigms such as four-plate test will be required in future experiment.

### Chronic High-Fat Diet-Induced Anxiety-Like Behaviors Are Weakened by GORZ Through Regulation of Dopamine Signaling

It has been well-documented that neural systems, including the hippocampus, nucleus accumbens, prefrontal cortex, and amygdala, can be altered by overconsumption of sugary and fatty foods, leading to poor mental health involved in cognition and anxiety (Avena et al., 2008; Morganstern et al., 2012; Murphy and Mercer, 2013; Bocarsly et al., 2015). Furthermore, the brain monoaminergic theory has been the prevailing hypothesis of anxiety, and is associated with altered brain functioning. It has been reported that DA and its metabolites, 3,4-dihydroxyphenylacetic acid and HVA, increase significantly in the cerebral cortex following a long-term HFD intake (Wakabayashi et al., 2015). Furthermore, it was also recently reported that a HFD was associated with sensitization of the DA mesolimbic pathway, with higher bursting activity of DA neurons and enhanced DA release, and greater expression of tyrosine hydroxylase and D2 receptors in the nucleus accumbens (Naneix et al., 2017). In the present study, the significantly increased levels of 5-HT, DA, and NE in the amygdala of the HFD group suggest that chronic HFD exposure for 8 weeks also triggered the synthesis of these brain levels of monoamines in the amygdala. Noticeably, GORZ significantly downregulated the increased DA in the amygdala but not in the cerebral cortex, indicating that GORZ may have regulated DA synthesis in an amygdala-specific manner in HFD-fed mice. Moreover, the amygdala's fundamental role in the regulation of anxiety has been underscored in many previous studies (Etkin et al., 2009; Tye et al., 2011; Akter et al., 2019). In the current study, we could not show any notable changes in the striatum levels of monoamines and their metabolites (see **Supplementary Figure 1**), although it is widely reported that the striatum is a brain area where the dopaminergic neurons in the substantia nigra project, in order to maintain DA homeostasis (Hu et al., 2004).

### The Possible Mechanisms of GORZ in the Regulation of Dopamine Signaling

The intricate mechanism of GORZ that underlies the regulation of DA functioning in the amygdala remains unclear. Potentially, G-coupled DA receptors [two classes: D1-like (e.g., D1, D5) and D2-like (e.g., D2, D3, D4)] may play an important role in this regulation. D2-like receptors (highly expressed in the striatum) (Beaulieu and Gainetdinov, 2011) inhibit adenylyl cyclase and decrease cAMP production by coupling with Gαi/o proteins (Missale et al., 1998; Beaulieu and Gainetdinov, 2011; Money and Stanwood, 2013). Tyrosine hydroxylase, the rate-limiting enzyme for DA synthesis (Labouesse et al., 2013) may be another possible target of GORZ treatment. It is possible that HFD-induced monoamine synthesis in brain regions preferentially occurs in the amygdala. Further investigation is indeed necessary to examine the detailed mechanisms by which GORZ regulates HFD-induced central monoamine synthesis and metabolic dysfunction.

### The Possible Antiinflammatory Mechanisms of GORZ in a Chronic HFD- Induced Anxious State

Inflammation is one of the main characteristics of excessive weight gain/obesity and affects the blood, peripheral systems, and the central nervous system (De Souza et al., 2005; Posey et al., 2009; Erion et al., 2014). Proinflammatory cytokines interact with multiple pathways known to influence mood regulation (Haroon et al., 2012). A study demonstrated that a HFD significantly increased the expression of proinflammatory cytokines in the hippocampus such as interleukin-6, IL-1β and TNF-α (Dutheil et al., 2016). We found that an 8-week chronic HFD intake increased vulnerability to anxiety-like behaviors. The anxiogenic effects of a high-fat diet in the current study were mediated by proinflammatory signaling, induced by chronic HFD consumption. Moreover, a recent study reported that brain inflammation was evident following the onset of HFD consumption, prior to any substantial weight gain (Thaler et al., 2012). In this regard, we detected significantly increased relative mRNA levels of proinflammatory cytokines *Tnf-α* in the amygdala—favorably so—upon effecting HFD treatment, suggesting that there is a link among HFD-induced anxiety-like behaviors and inflammation. Interestingly, and more importantly, upregulation of these proinflammatory cytokines in the amygdala was actively blocked in HFD-fed mice receiving a 0.5% GORZ supplementary diet for 8 weeks, indicating an antineuroinflammatory response in these mice.

It is well-known that the activation of nuclear factor NF-kappa-B (NFKB) triggers the proinflammatory signaling pathway, leading to overproduction of cytokines by macrophages (Sakai et al., 2012). GORZ may downregulate the expression of inflammatory transcription factors in macrophages, such as toll-like receptors (TLR-2 and TLR-4) and NFKB p65 subunit (Rao et al., 2016), which in turn may decrease the expression of proinflammatory cytokines such as *Tnf-α* and *Il-1* (Barbu et al., 2009).

At present, we do not have any information whether GORZ directly interact with any receptor, transporter or enzymes, however we are attentive to get any information on the protein with which GORZ interact.

**Figure 7:**
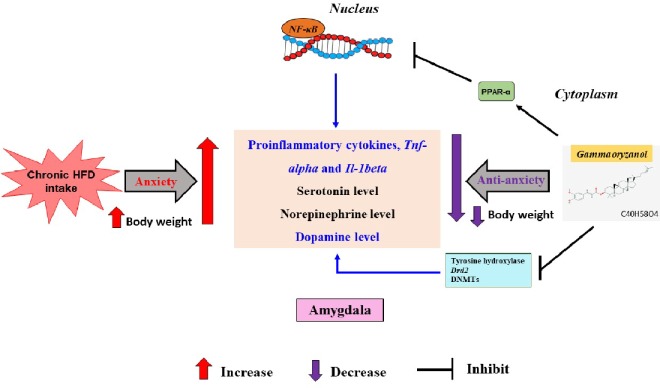
The possible brain monoaminergic and antiinflammatory pathways underling the anxiolytic effect of 0.5% GORZ in the HFD-induced anxiety mice model.

Recently, intact GORZ, along with its metabolites, were detected in mice plasma, brain and peripheral organs after long time feeding of GORZ (Kobayashi et al., 2019). The concentrations of GORZ and metabolites in brain were 100–1,000 times lower than those in the peripheral organs, suggesting the possible mechanism that GORZ would act in periphery firstly and then act in the brain. Direct injection of GORZ into brain is next required experiment to show direct effect of GORZ on brain function. Clear dose-dependency study of GORZ (both *in vivo* and *in vitro*) would be more beneficial for brain function. Based on aspects of the above discussion, the possible brain monoaminergic and antiinflammatory pathways involved in the improvement of HFD-induced anxiety-like behavior following GORZ feeding is illustrated in **Figure 7**. However, we could not directly demonstrate that dopamine, TNF-α and IL-1β are involved in the anxiolytic-like effects of GORZ.

In the current experiment, we evaluated the anxiolytic-like effects of 0.5% GORZ by comparing only three groups, i.e., a control, HFD, and HFD + GORZ. However, in future experiments, we must include standard anxiolytic agents (benzodiazepines) to represent a fourth group, in order to observe relative effects. Further clinical experiments are also needed to include clinically prevalent concepts and ideas about obesity-induced neuropsychiatric diseases to provide further insights.

In conclusion, the data presented herein support the potential use of a dietary 0.5% GORZ for designing an innovative therapeutic approach for preventing HFD-induced anxiety-like behaviors, and associated ambiguities such as central dopaminergic dysfunction and inflammation.

## Data Availability Statement

The raw data supporting the conclusions of this article will be made available by the authors, without undue reservation, to any qualified researcher.

## Ethics Statement

All the experimental animal protocol and procedures were carried out in accordance with the “Fundamental Guidelines for Proper Conduct of Animal Experiment and Related Activities in Academic Research Institutions” (published by the Ministry of Education, Culture, Sports, Science and Technology, Japan) and approved by the Committee for Animal Experimentation of the School of Science and Engineering at Waseda University (permission #2017-A074).

## Author Contributions

SA and SS designed the study. SS also supervised the project. SA and KU performed experiments. SA, KU, and HS performed the sampling. SA and HS analyzed the data. SA wrote the paper. Consents are taken from every author.

## Funding

This work was partially supported by the Council for Science, Technology, and 33 Innovation and the cross- ministerial Strategic Innovation Promotion Program, and Japan 34 Society for the Promotion of Science (JSPS) KAKENHI (A and Houga) [Shibata. S].

## Conflict of Interest

The authors declare that the research was conducted in the absence of any commercial or financial relationships that could be construed as a potential conflict of interest.

## Abbreviations

cAMP, cyclic adenosine monophosphate; DA, dopamine; EPM, elevated plus maze; GORZ, γ–oryzanol; HFD, high-fat diet; HPLC-ECD, high-performance liquid chromatography-electrochemical detection; HVA, homovanillic acid; IL-1 β, interleukin-1 beta; MHPG, 3-methoxy-4-hydroxyphenylglycol; NE, norepinephrine; NFKB, nuclear factor NF-kappa-B; OFT, open-field test; RT-PCR, real-time reverse transcription-polymerase chain reaction; TLRs, toll-like receptors; TNF-α, tumor necrosis factor-alpha; ZT, Zeitgeber time; 18S rRNA, 18S ribosomal RNA; 5-HIAA, 5-hydroxyindole acetic acid; 5-HT, 5-hydroxytryptamine.
