# Supplementary material for: Gamma Oryzanol Alleviates High-Fat Diet-Induced Anxiety-Like Behaviors Through Downregulation of Dopamine and Inflammation in the Amygdala of Mice
Source: Front Pharmacol. 2020 Mar 17;11:330. doi: 10.3389/fphar.2020.00330 (PMC7090127; doi:10.3389/fphar.2020.00330)
Supplement: Supplementary file 1 [file Table_1.docx]

| **Gene** |  | **Sequences** |
| --- | --- | --- |
| ***Tnf-*** | Forward  Reverse | 5´-gagcacagaaagcatgatcc -3´  5´-ccacaagcaggaatgagaag -3´ |
| ***Il-1β*** | Forward  Reverse | 5′-tccatgagctttgtacaagg -3′  5′-ggtgctgatgtaccagttgg -3′ |
| ***18srRNA*** | Forward  Reverse | 5´-gggcagtatggttgcaaagc -3´  5´-tgtcaatcctgtccgtgtcc -3´ |

**Supplemental Table 1**　 Primer sequence for reverse transcription polymerase

chain reaction
