# Supplementary figures and images for: Gamma Oryzanol Alleviates High-Fat Diet-Induced Anxiety-Like Behaviors Through Downregulation of Dopamine and Inflammation in the Amygdala of Mice
Source: Front Pharmacol. 2020 Mar 17;11:330. doi: 10.3389/fphar.2020.00330 (PMC7090127; doi:10.3389/fphar.2020.00330)

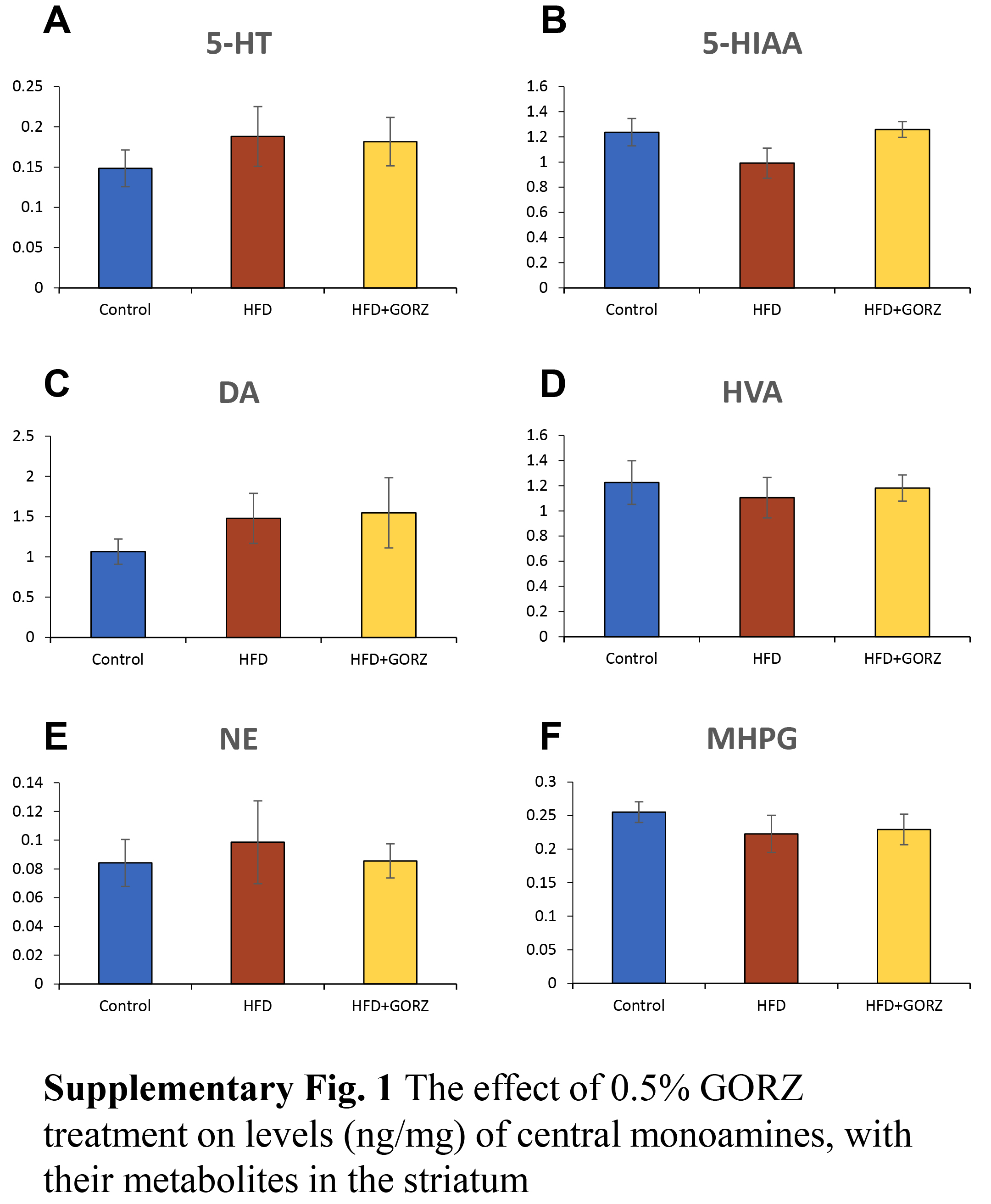

Supplement: Supplementary file 2 [file Image_1.jpg]
